# A Case of Stevens–Johnson Syndrome in Recurrent Late-Stage Ovarian Cancer Patient after Management of Chronic Pain with Elastomeric Pump

**DOI:** 10.3390/curroncol28040256

**Published:** 2021-08-03

**Authors:** Andrej Cokan, Vida Gavrić Lovrec, Iztok Takač

**Affiliations:** 1 Department for Gynaecological and Breast Oncology, University Medical Centre Maribor, Ljubljanska ulica 5, 2000 Maribor, Slovenia; 2 Medical Faculty, University of Maribor, Taborska ulica 8, 2000 Maribor, Slovenia; vida.gavric@ukc-mb.si (V.G.L.); iztok.takac@ukc-mb.si (I.T.); 3 Department for Reproductive Medicine and Gynaecological Endocrinology, University Medical Centre Maribor, Ljubljanska ulica 5, 2000 Maribor, Slovenia; 4 Clinic for Gynaecology and Perinatology, University Medical Centre Maribor, Ljubljanska ulica 5, 2000 Maribor, Slovenia

**Keywords:** ondansetron, ovarian cancer, Stevens–Johnson syndrome

## Abstract

(1) Background. Stevens–Johnson syndrome (SJS) and toxic epidermal necrolysis (TEN) are severe mucocutaneous reactions, characterized by extensive necrosis and detachment of the epidermis. (2) Case presentation. We present a case of a 46-year-old patient with late-stage high-grade serous ovarian cancer who was primarily treated with neoadjuvant chemotherapy and interval debulking, which was followed by adjuvant chemotherapy. At first recurrence, she was again treated with chemotherapy, and due to severe abdominal pain, an elastomeric pump containing analgesics, anti-inflammatories, and ondansetron was administered. In the same month, she was admitted to the hospital due to severe dysphagia, and in the following days she developed haemorrhagic vesiculobullous lesions on the facial skin and trunk. Stevens–Johnson syndrome was confirmed and ondansetron as a plausible leading cause was discontinued. Despite multimodal treatment, her condition deteriorated, and she died. (3) Discussion and conclusion. Although gynaecologists rarely encounter Stevens–Johnson syndrome, high mortality of the disease should ensure a low threshold for diagnosing and treating this disease.

## 1. Introduction

Stevens–Johnson syndrome (SJS) and toxic epidermal necrolysis (TEN) are severe mucocutaneous reactions that are considered a delayed-type hypersensitivity reaction to medication and are characterized by extensive necrosis and detachment of the epidermis [1]. Both are rare diseases, affecting 1 to 6 and 0.4–1.2 per million people each year, respectively [2], and are uncommon in the field of gynaecological oncology. They are associated with a wide array of medications, ondansetron being a possible but quite rare cause of the disease [3]. Treatment of this condition requires a multidisciplinary approach and consists of supportive treatment, local skin and mucosal membranes care, ocular management, and different adjunctive therapies. The SCORe of Toxic Epidermal Necrosis (SCORTEN) scale is used for predicting and assessing mortality rate for a certain patient based on independent risk factors. Mortality rate is high despite all treatment options and is higher in cancer patients.

## 2. Case Presentation

We present a 46-year-old patient with late-stage high-grade serous ovarian cancer with SJS, presumably due to therapy with ondansetron.

**Figure 1:**
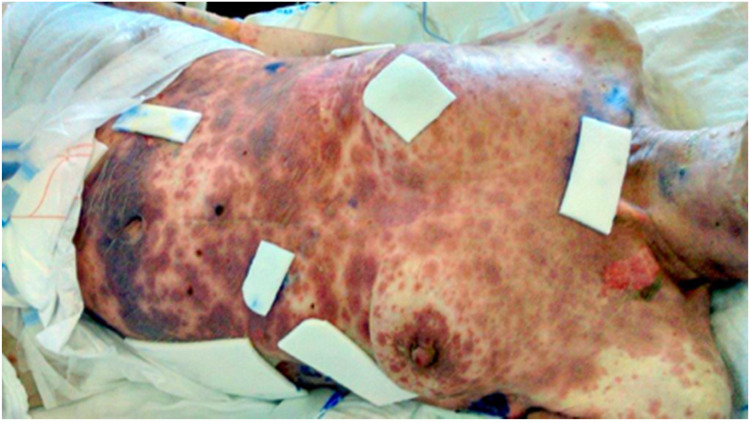
Haemorrhagic vesiculobullous lesions on facial skin and trunk.

She first came to our clinic in April 2018 with clinical and ultrasonographic signs of ovarian cancer. Diagnostic laparoscopy with biopsy confirmed high-grade serous ovarian cancer (p53+, ER+ in 90%, PR-, WT-1+, CK7+, p53+). The disease was primarily inoperable, FIGO stage IVA, so neoadjuvant chemotherapy was suggested. After five cycles of paclitaxel with carboplatin, the extent of the disease was re-evaluated. Adequate regression of the disease on CT scans was observed, so we performed an interval cytoreduction (total abdominal hysterectomy with bilateral salpingo-oophorectomy, omentectomy, appendectomy, resection of parts of small bowel, and sigma with the formation of the stoma). Surgery was not radical, with remaining lesions on the right and left diaphragm. After surgery, patient received two more cycles of paclitaxel with carboplatin. Treatment was completed in January 2019 when the patient was offered to continue treatment with bevacizumab, which she refused. The first recurrence was diagnosed in July 2019 with elevated Ca125 and with CT scans showing enlarged retroperitoneal lymph nodes, a lesion in the right pulmonary lobe, and enlarged pericardial lymph nodes. She became symptomatic in September 2019 when she presented with ascites. We offered her chemotherapy round 2 with carboplatin and liposomal doxorubicin. During this treatment, she experienced severe abdominal pain, which was treated predominantly with morphine. Left side hydronephrosis also developed and was treated with percutaneous nephrostomy. After receiving seven cycles of adjuvant chemotherapy, due to a BRCA positive test result, olaparib was offered but she again refused the proposed treatment. To relieve severe abdominal pain, an elastomeric pump was implanted at the beginning of August 2020 containing morphine, esketamine, dexamethasone, xylocaine, and ondansetron. At the end of August 2020, the patient was admitted to the hospital because of elevated body temperature, dysphagia, and difficulty breathing. She also had redness and oedema of the pharynx and periocular inflammation. Acute pharyngitis was suspected and Co-amoxiclav (1.2 g IV tid) was introduced. After two days, round red lesions, macules, and plaques, which were present in the periocular region and on the trunk, started emerging, so she was examined by an infectious disease specialist and dermatologist. Laboratory findings included elevated CRP, PCT, bicytopenia with monocytic predominance, electrolyte disbalance, hypoalbuminemia, and normal renal function. Her HIV status was unknown. Atypical pneumonia (immunology results later showed elevated IgG for Mycoplasma pneumoniae) and SJS were suspected, so antibiotic treatment was changed to moxifloxacin (400 mg IV qd) and flucloxacillin (2 g IV qid) and we also introduced methylprednisolone (40 mg IV qd) with pantoprazole (40 mg IV qd) and local therapy for severely affected skin on the trunk (betamethasone lotion), vulva (betamethasone/gentamicin lotion), around the lips (triamcinolone), and eyes (chloramphenicol lotion for the left eye and dexamethasone/neomycin/polymyxin B lotion for the right eye). Ondansetron as a most likely cause of the syndrome was discontinued (Naranjo Adverse Drug Reaction Probability Scale = 2), but despite that, skin lesions deteriorated with the additional appearance of haemorrhagic vesiculobullous lesions, especially on the facial skin, which we treated with silicone and polyurethane patches and sodium fusidate (Figure 1). A SCORTEN score of 3 was calculated. Analgesic therapy (PCA pump with 80 mg of morphine, 50 mg of cetanest, 10% lidocaine with up to 1000 mL of saline solution), electrolytes, fluids, and total parenteral nutrition were administered. On the fifth day of treatment, the extent of epidermal detachment was approximately 8–10%. CRP was still elevated, and elevated body temperature rose to 39 °C. The antibiotic regimen was changed to piperacillin/tazobactam (4.5 g IV tid), which resulted in the drop of inflammatory markers, although the general condition of the patient did not improve. In the following days, we observed deterioration of liver function and thrombocytopenia. The extent of epidermal detachment increased since a majority of the skin on the trunk was affected. Additionally, due to less controllable pain because of severe skin lesions with exposure of deeper skin layers, we had to modify analgesic therapy with the addition of midazolam and higher doses of morphine. Despite all efforts of a multidisciplinary team (infectious disease specialists, dermatologists, ophthalmologists, gynaecologists, and plastic surgeons) the patient’s general condition further deteriorated, and on the 12th day after being admitted to the hospital she died. 

Beforehand, written consent was obtained from the patient allowing publication of the case report and all accompanying graphic material. 

## 3. Discussion

SJS/TEN is a very serious drug-adverse mucocutaneous reaction with a high mortality rate despite all available treatment options. Both SJS and TEN are rare, SJS being three times more common than TEN, and the joint incidence is about 1–7/1,000,000 per year. The incidence is higher in immunodeficient and cancer patients and women [2]. Medications, such as allopurinol, lamotrigine, sulfasalazine, nevirapine, nonsteroidal anti-inflammatory drugs, phenobarbital, carbamazepine, paracetamol, penicillin such as amoxicillin or ampicillin, and various anticancer drugs are thought to be the leading triggers for developing the disease [4,5]. Treatment is multidisciplinary and consists of supportive care (wound care, fluids and nutrition [6], pain control, prevention and treatment of infections, and prevention of vulvovaginal sequelae), ocular management, and different adjunctive therapies (systemic corticosteroids, intravenous immune globulin, cyclosporine, thalidomide, tumour necrosis factor inhibitors, and plasmapheresis) [7,8,9,10]. Despite multiple treatment options, mortality is still high and can range from 10% for SJS to up to 30% or more for TEN [11]. Acute respiratory distress syndrome, sepsis, and multiple organ failure are the most common causes of in-hospital death. Patients above 70 years of age with comorbidities, such as metastatic cancer or liver diseases, are also associated with an increased risk of death [12]. 

As a prediction score, the SCORTEN scale or score is used widely by clinicians to determine which clinical setting is appropriate for the management of the individual patient and for discussing a patient’s prognosis [13] [Table 1]. The SCORTEN scale consists of seven independent risk factors for high mortality, the presence of which is scored with one point for each present and interpreted as follows: age ≥ 40 years; heart rate ≥ 120 beats per min; cancer/hematologic malignancy; body surface area detached ≥ 10% (at day 1); serum blood urea nitrogen > 10 mmol/L; serum bicarbonate < 20 mmol/L; serum glucose > 14 mmol/L.

Although ovarian neoplasm or more specifically a treatment for ovarian neoplasm and ondansetron, on the other hand, have been mentioned separately as a possible cause for the development of the disease, both causes are very rare [14]. As gynaecologists, we are rarely subjected to recognizing and treating these diseases, which are also more fatal in our cancer patients, probably due to their immunocompromised state [15]. 

In our case, the patient already completed cancer-specific treatment and at the time she presented with the first symptoms, which then progressed into the SJS, did not receive any cancer-specific therapy. Additionally, different antibiotic regimens were applied slightly after the first onset of symptoms, so ondansetron, which was prescribed in an elastomeric pump to alleviate sickness due to opioid therapy, was suspected to be the most probable cause for the onset of the disease, although we have to comment that due to Naranjo Scale 2, possible previous infection with Mycoplasma pneumoniae (elevated IgG), and antibiotic treatment, it is difficult to be certain that ondansetron was truly the most probable cause for the onset of the disease. Despite establishing the diagnosis early, identifying the most probable cause for the disease development, and applying multimodality treatment, she deteriorated rapidly. We calculated her SCORTEN score (3), which meant that her risk of dying was around 35%. In the following days, the percentage of epidermal detachment increased from below 10% to the majority of skin on the trunk, which made it difficult to distinguish between SJS and TEN, so in this case the term severe cutaneous adverse reaction would be perhaps more appropriate. In addition, the patient developed sepsis and liver failure and finally died only 12 days after being admitted to the hospital. 

## 4. Conclusion

Although SJS/TEN is quite rare in gynaecological cancer patients, we should be aware that especially in ovarian cancer patients, who are exposed to prolonged multimodality treatment with surgery, chemotherapy, and biological agents and treatment can be accompanied by various signs and symptoms, such as infections, chronic pain, malaise, vomiting, and obstipation, which are treated with multiple medications, the occurrence of this disease is possible and is associated with very high morbidity and mortality.

## Figures and Tables

**Table 1:** SCORTEN score interpretation of the results.

Number of Risk Factors	Mortality Rate
0 to 1	3.2%
2	12.1%
3	35.3%
4	58.3%
5 or more	>90%

## Data Availability

No new data were created or analysed in this study. Data sharing is not applicable to this article.

## References

[1] Stern R.S., Divito S.J. (2017). Stevens-Johnson Syndrome and Toxic Epidermal Necrolysis: Associations, Outcomes, and Pathobiology—Thirty Years of Progress but Still Much to Be Done. J. Investig. Dermatol..

[2] Nayak C.S., Pereira R.R., Dhurat R.S., Saraogi P.P. (2012). Inadvertent provocative oral ondansetron use leading to toxic epidermal necrolysis in an HIV-infected patient. Indian J. Dermatol..

[3] Sekula P., Dunant A., Mockenhaupt M., Naldi L., Bavinck J.N.B., Halevy S., Kardaun S., Sidoroff A., Liss Y., Schumacher M. (2013). Comprehensive Survival Analysis of a Cohort of Patients with Stevens–Johnson Syndrome and Toxic Epidermal Necrolysis. J. Investig. Dermatol..

[4] Collins L.K., Chapman M.S., Carter J.B., Samie F.H. (2017). Cutaneous adverse effects of the immune checkpoint inhibitors. Curr. Probl. Cancer.

[5] Paulmann M., Mockenhaupt M. (2017). Fever in Stevens–Johnson Syndrome and Toxic Epidermal Necrolysis in Pediatric Cases: Laboratory Work-up and Antibiotic Therapy. Pediatr. Infect. Dis. J..

[6] Creamer D., Walsh S., Dziewulski P., Exton L., Lee H., Dart J., Setterfield J., Bunker C., Ardern-Jones M., Watson K. (2016). U.K. guidelines for the management of Stevens–Johnson syndrome/toxic epidermal necrolysis in adults 2016. Br. J. Dermatol..

[7] Chang Y.-S., Huang F.-C., Tseng S.-H., Hsu C.-K., Ho C.-L., Sheu H.-M. (2007). Erythema multiforme, Stevens-Johnson syndrome, and toxic epidermal necrolysis: Acute ocular manifestations, causes, and management. Cornea.

[8] Huang Y.-C., Li Y.-C., Chen T.-J. (2012). The efficacy of intravenous immunoglobulin for the treatment of toxic epidermal necrolysis: A systematic review and meta-analysis. Br. J. Dermatol..

[9] Ng Q.X., De Deyn M.L.Z.Q., Venkatanarayanan N., Ho C.Y.X., Yeo W.-S. (2018). A meta-analysis of cyclosporine treatment for Stevens–Johnson syndrome/toxic epidermal necrolysis. J. Inflamm. Res..

[10] Wang C.-W., Yang L.-Y., Chen C.-B., Ho H.-C., Hung S.-I., Yang C.-H., Chang C.-J., Su S.-C., Hui R.C.-Y., Chin S.-W. (2018). Randomized, controlled trial of TNF-α antagonist in CTL-mediated severe cutaneous adverse reactions. J. Clin. Investig..

[11] Mockenhaupt M., Bastuji-Garin S., Auquier-Dunant R., Ziemer L. (2011). The current understanding of Stevens–Johnson syndrome and toxic epidermal necrolysis. Expert Rev. Clin. Immunol..

[12] Ezaldein H., Totonchy M., Chow C., Samuel A., Ventura A. (2017). The effect of comorbidities on overall mortality in Stevens- Johnson Syndrome: An analysis of the Nationwide Inpatient Sample. Dermatol. Online J..

[13] Torres-Navarro I., Briz-Redón Á., Botella-Estrada R. (2020). Accuracy of SCORTEN to predict the prognosis of Stevens-Johnson syndrome/toxic epidermal necrolysis: A systematic review and meta-analysis. J. Eur. Acad. Dermatol. Venereol..

[14] Giaccone G., Risio M., Bonardi G., Calciati A. (1986). Stevens-Johnson Syndrome and Fatal Pulmonary Toxicity to Combination Chemotherapy Containing Bleomycin: A Case Report. Tumori J..

[15] Gillis N.K., Hicks J.K., Bell G.C., Daly A.J., Kanetsky P.A., McLeod H.L. (2017). Incidence and Triggers of Stevens-Johnson Syndrome and Toxic Epidermal Necrolysis in a Large Cancer Patient Cohort. J. Investig. Dermatol..

